# Liquid Penetration Depth and Strength of Concretes Modified with Polymer Admixtures Under the Action of Crude-Oil Products

**DOI:** 10.3390/ma12233900

**Published:** 2019-11-26

**Authors:** Maciej Gruszczyński, Małgorzata Lenart

**Affiliations:** Faculty of Civil Engineering, Cracow University of Technology, 31-155 Cracow, Poland; mgruszczynski@pk.edu.pl

**Keywords:** waterproof concrete, polymer admixtures, crude-oil products, light liquids, depth penetration of liquids

## Abstract

In the present article, the research results of concretes modified with a polymer dispersion of vinyl-benzene and acrylic (PC1) and with a styrene–butadiene dispersion (PC2) are discussed. Concretes were exposed to diesel, non-ethylated fuel, and the standard mixture of light liquids for 1000 h. Concretes modified with polymer dispersions, especially with the styrene–butadiene dispersion (PC2), indicated a smaller degree of liquid penetration into the depth of samples compared to the control concrete. The compressive strength for tested concretes, determined after 1000 h of storage in crude-oil products, in comparison to the strength of samples stored in de-mineralized water was significantly decreased, with the maximum differences equal to 12% for PC2 concrete.

## 1. Introduction

Currently, concrete constitutes the most frequently applied structural material. It is used in various conditions, including in environments subjected to the action of crude-oil products as well as so-called “light liquids”. Light liquids are generally organic compounds derived from the processing of crude oil. These substances are used in various areas of the economy, including as fuels and lubricants. According to the definition given in PN-EN 858-1, a light liquid means a liquid with a density that does not exceed 0.95 g/cm^3^, which means it is practically insoluble and does not saponify. Unfortunately, these substances pollute our surrounding environment [1] and are one of the most serious environmental problems. Soil and water are particularly at risk. Crude-oil products or oils inhibit the self-purification process of water and reduce the absorption of oxygen [2]. The main sources of pollution for these substances are oil mining, gas stations, car workshops and car washes, transport, and sewage polluted with light substances. Structures that include such types of materials are industrial floors [3,4], floors in car repair shops and washes, surface concrete for roads [5] and airports [6], as well as the structures in sewage treatment plants, e.g., separators [7]. The task of separators is to separate crude-oil products and prevent their escape to sewer systems and penetration into groundwater.

Concrete and reinforced concrete structures working in such aggressive environmental conditions must have adequate durability, reaching at least 50 years. Requirements concerning the durability of concrete are formulated in PN-EN 206, while those for reinforced concrete structures are specified in PN-EN 1992-1-1.

Long-term exposure to crude-oil products destructively influences the properties of concrete [8,9,10,11,12] and, consequently, the behavior of reinforced concrete structures [13,14,15,16]. The compressive strengths of normal concrete and high-strength concrete under exposure to light liquids are reduced, with normal-strength concrete having about a 10% higher reduction than high-strength ones [17]. This is connected to the higher tightness of high-strength concretes and the resulting lower susceptibility to penetration by crude-oil products. In research, a higher reduction in concrete strength subjected to the action of liquids with lower viscosity has also been observed [17,18,19]. During conducted tests, the influence of mineral oil on the long-term strength of normal-strength and high-strength concretes has been documented [15,20,21,22]. After 280 days, the compressive strength of concrete stored in oil was about 17% lower (12% for high-strength concrete) than the control concrete (matured in water). However, in comparing the strength after 280 days with values obtained after 28 days for concretes subjected to mineral oil storage, no significant differences in strength were noticed due to the slowing down of, or even stopping of, the cement hydration reactions. Within these tests, it was also noted that the degree of saturation of concrete with oil, as well as the concrete’s porosity and permeability [23], significantly influenced the strength reduction level.

Tests conducted so far have indicated that crude-oil products affect the concrete physically and chemically, causing the loosening of the contact zone between the aggregate and paste as well as slowing down or even stopping the cement hydration processes [24,25]. The mechanism of concrete destruction under the influence of crude-oil products is of a biological and chemical nature because of the combined interaction of bacteria contained in these substances and the chemical impact of the compounds they produce. In the crude-oil water system, there are both aerobic and anaerobic bacteria [26], and these microorganisms cause a change in the properties of these liquids. The products of these reactions can be organic acids (e.g., acetic acid) as well as inorganic acids (e.g., sulfuric acid), and they also cause a decrease in the pH of the liquid [3]. Concrete dissolves and softens in such an environment, which increases its porosity. Such deterioration results in the destruction of the contact between the concrete and reinforced steel in reinforced concrete structures [27].

Due to the destructive impact that crude oil has on concrete and the natural environment that surrounds us, a program of testing concrete modified with polymers has been prepared and carried out. The aim of the present research was to determine the influence of the action of crude-oil products (i.e., diesel and non-ethylated fuel) as well as light liquids on the mechanical (compressive strength) and physical properties (water absorption, liquid penetration depth, and freeze–thaw resistance) of concretes modified with polymers. An admixture of polymers causes the formation of a non-continuous membrane on cement grains [28,29], which may result in the impediment of water and other liquids in accessing cement grains, thus increasing the resistance of these concretes to light liquids in the environment.

## 2. Materials and Methods 

The testing program included preparation of three types of concrete:

- Control concrete (CC);
- Concrete modified with a polymer dispersion of vinyl-benzene and acrylic (PC1); and
- Concrete modified with styrene–butadiene dispersion (PC2).

Initially, a concrete strength class of C35/45 and a water-tightness degree of W8 were assumed, as is typical for surface concrete used for petrol stations. For practical reasons, a concrete mix with a consistency of S3–S4 was applied. For this purpose, super-plasticizer based on poli-carbo-xylan was added to the mix of the control concrete (CC) and the PC1 mix. No super-plasticizer was applied for the PC2 mix, as the dispersion that was used indicated the fluxing action. All concretes were prepared following Portland cement CEM I 42,5 N-SR3/NA guidelines destined for structures subjected to aggressive environments, in which the concrete is characterized by a low alkali content (≤0.60% expressed as Na_2_O_eq_) and sulfate resistance (for SR3, tricalcium aluminate content in C_3_A clinker ≤3%, additionally, the sum of C_4_AF + 2xC_3_A is <20%). The chemical composition and physical properties of the cement are shown in Table 1. Natural sand with a 0/2 fraction was applied together with coarse granite aggregate with fractions of 2/8 and 8/16. The concrete mix compositions are presented in Table 2. Concrete cubic samples (150 mm) were then formed from the prepared mixes. All samples, after being taken out of the mold, were stored in water for 28 days, in compliance with the requirements specified in PN-EN 12390-2.

The following tests were carried out within the research program:

- Consistency measurements for the concrete mix determined with the cone fall method, according to PN-EN 12350-2;
- Determination of the concrete mix density, according to PN-EN 12350-6;
- Determination of the compressive strength of concrete cubic samples, according to PN-EN 12390-3, tested after 28 days of hydration;
- Water absorption measurements in compliance with PN-B 06250:1988, tested after 28 days of hydration;
- Determination of the water permeability for concrete, according to PN-B 06250:1988, tested after 28 days of hydration;
- Determination of the compressive strength after storing the samples in crude-oil products, according to PN-EN 858-1 and PN-EN 12390-3, tested after 28 days of hydration plus 1000 hours of storing samples in crude-oil products; and
- Measurements of the penetration depth of non-ethylated fuel into concrete samples after a specified time, conducted by splitting the samples and measuring the depth of fuel penetration and tested after 28 days of hydration plus 1000 h of storing samples in crude-oil products.

The aims of the fresh concrete tests were to determine the effect of adding the polymer admixture on the consistency and density of the mix (i.e., to determine whether there are no side effects in the form of aeration of the fresh concrete). The water absorption and water permeability tests were determined in accordance with the procedures set out in the Polish standard PN-B 06250.

The water absorption test was carried out on three samples of regular shapes for each type of concrete. The samples were gradually saturated with water up to a constant weight, then they were dried in a drier at 105 °C to a constant weight, and their water absorption was calculated. In accordance with the requirements given in this standard, the absorption of concrete should not be greater than 5% in the case of concrete being directly exposed to external weathering.

Furthermore, tests of water permeability through concrete were carried out on six cubic or cylindrical samples. The surface of hardened cement grout was roughened on a 100 mm diameter circle on the wall which was to be affected by water. The remaining surfaces were covered with a waterproof coating. The samples were fixed in the apparatus, loaded with water at a pressure of 200 kPa, and the pressure was increased by another 200 kPa every 24 h. The test ended when three of six samples showed signs of leakage or when the required degree of water resistance was achieved. For example, a W8 degree of water resistance means that the samples were exposed to water at a pressure of 800 kPa for a minimum of 24 kPa, and the samples showed no signs of leakage at that time. The samples were split to determine the depth of water penetration into the concrete after testing.

Other tests were carried out in accordance with the requirements of the European standards listed above.

## 3. Test Results and Analysis 

### 3.1. Fresh Concrete Tests

Test results for the consistency and density of individual concrete mixes are presented in Table 3. The consistency, determined by a slump test, was 150 mm for control concrete, while for PC1 and PC2 concrete it was 170 mm and 165 mm, respectively. In compliance with the assumptions made, the consistency was kept on the boundary between classes S3 and S4.

The density for all mixes was equal to around 2400 kg/m^3^ (Table 3). No aeration effect was observed with the combination of polymeric admixtures and super-plasticizer.

### 3.2. Tests of Hardened Concrete After 28 Days of Hydration

After 28 days of sample hydration in water, tests were conducted for compressive strength, which were carried out on three cubic samples for each type of concrete. The obtained results for the compressive strength after 28 days of hydration are shown in Table 4.

After 28 days of concrete maturation in water, the compressive strengths oscillated within the range of 50–65 MPa. The mean compressive strength for control concrete (CC) was equal to 62 MPa. Both polymer-modified concretes indicated slightly lower strengths: 53.5 MPa for concrete PC1 and 55.5 MPa for concrete PC2. All concretes were designed and executed with the same w/c ratio; however, modification of cement composite structures with polymers may cause a slight (up to a few percent) reduction in the compressive strength because of the difficult access of water to cement grains [22,23]. This decrease was less than 14% for PC1 concrete and about 10% for PC2 concrete in relation to the control concrete.

**Figure 1:**
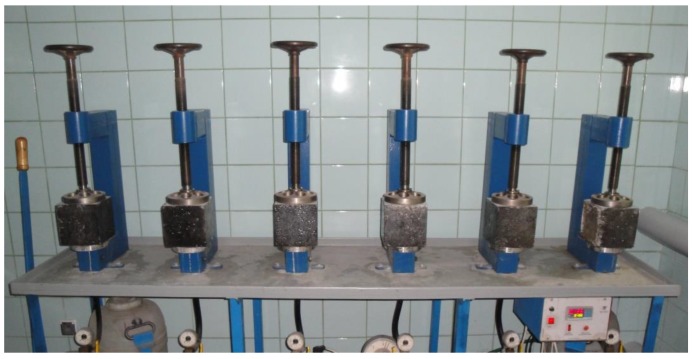
The measuring setup for the water permeability test.

Moreover, measurements of water absorption and permeability were made according to PN-B 06250. Water absorption was determined for three cubic samples with dimensions equal to 150 mm, while permeability was determined on six samples for each concrete type. The water permeability test was stopped after reaching the water pressure corresponding to the W8 water-tightness class. Then, the samples were split, and the depth of penetration of water into the sample was measured. Obtained results for water absorption and permeability are summarized in Table 5. Figure 1 presents a view of concrete samples during the water permeability test.

All concretes had very low water absorption values: 4.0% for control concrete, 2.5% for the concrete modified with styrene–butadiene polymer, and 3.3% for the concrete modified with vinyl-benzene and acrylic polymer. The obtained values were less than the value of 5% required by PN-B 06250 for concretes exposed to external factors.

The depths of penetration for water acting under a pressure of 800 kPa (required for the assumed W8 water resistance level) were 15 mm and 30 mm, respectively, for the styrene–butadiene polymer and vinyl-benzene and acrylic polymer concretes. These values correspond to a high level of water-tightness for concretes exposed to an aggressive environment.

### 3.3. Tests of Hardened Concrete After 1000 Hours of Storage in Light Liquids

Later in the research program, samples of individual concrete were subjected to resistance tests with crude-oil products and light liquids according to the procedure included in the code PN-EN 858-1. In compliance with this, concrete samples were stored for 1000 h in diesel in accordance with PN-ISO 8217 at a temperature of 23 ± 2 °C, non-ethylated fuel according to PN-EN 228 also at the temperature 23 ± 2 °C, as well as in a liquid mixture in accordance with point 8.1.4.1 of the code PN-EN 858-1 at a temperature of 40 ± 2 °C. The liquid mixture consisted of:

- 90% (m/m) de-mineralized water;
- 0.75% (m/m) sodium hydroxide;
- 3.75% (m/m) sodium orthophosphate;
- 0.50% (m/m) (meta) sodium silicate;
- 3.25% (m/m) sodium carbonate; and
- 1.75% (m/m) sodium metaphosphate.

**Figure 2:**
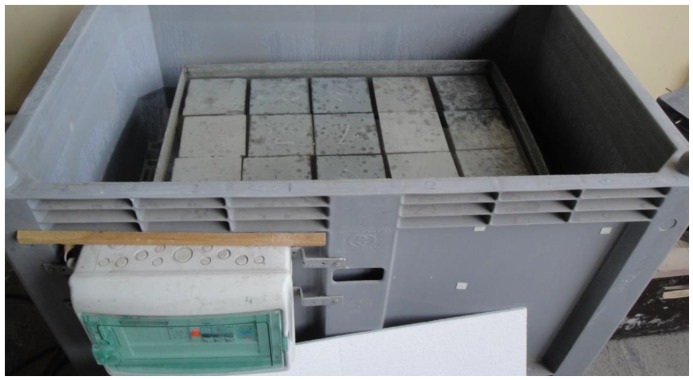
Samples stored in de-mineralized water at 40 °C.

**Figure 3:**
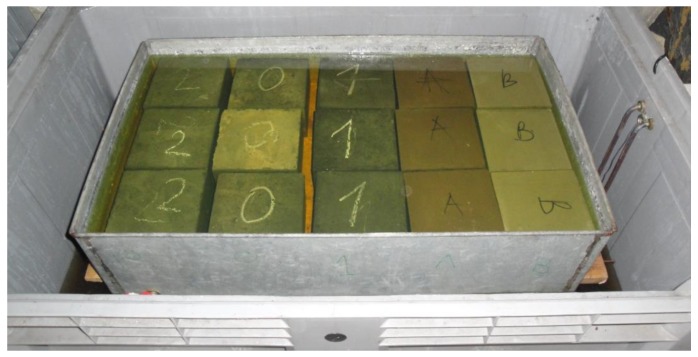
Samples stored in non-ethylated fuel at 23 °C.

**Figure 4:**
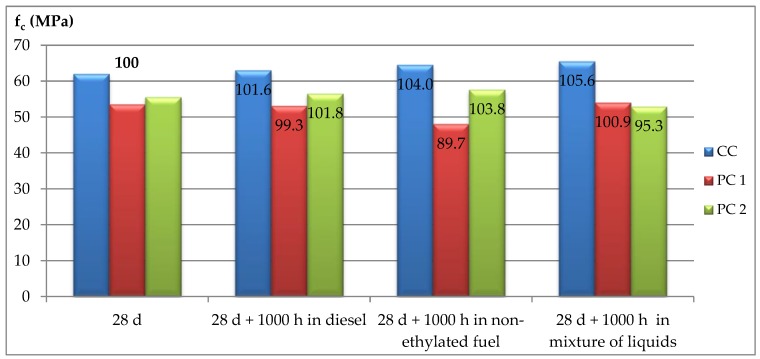
Compressive strengths of the tested concretes compared to the 28-day strength (numbers represent the percentage of the given strength in relation to the strength determined after 28 days).

**Figure 5:**
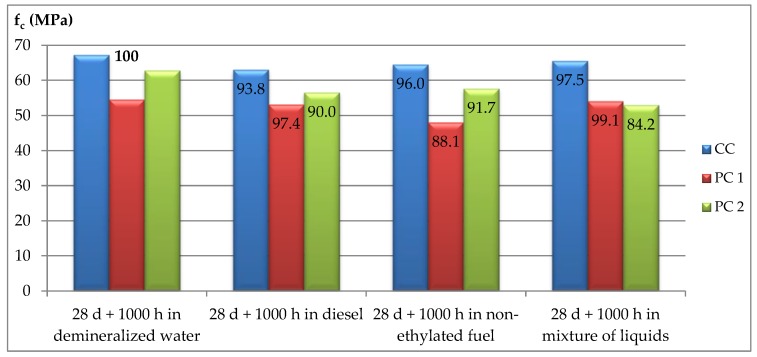
Compressive strengths of the tested concretes compared to storage conditions (numbers represent the percentage of the given strength in relation to the strength determined after 28 days).

In pursuance of specifying this procedure, a part of each sample was stored as a control in de-mineralized water at a temperature of 40 ± 2 °C. After this, the samples were taken out of the water and rinsed and dried in air for 24 h at a temperature equal to 20 °C. Then, the compressive strengths for individual samples were determined. The samples stored in water are shown in Figure 2, and those stored in a non-ethylated fuel are shown in Figure 3. The summary results of strength tests for particular concretes as well as storage conditions are given in Table 6 and in Figure 4 and Figure 5.

As the results show, after 1000 h of storage in de-mineralized water at a temperature of 40 °C, samples indicated further increases in strength for each concrete in comparison to the 28-day strength.

From analyzing the results obtained for the different concretes, it may be stated that the strengths of the concretes after storage in crude-oil products and those in the standard mixture of liquids had comparable 28-day strengths. The only exception was for the PC1 concrete, which, after storage in non-ethylated fuel, indicated about a 10% decrease in strength compared to the 28-day value.

In comparing the strengths of the tested concretes after storage in crude-oil products and in de-mineralized water, there was a reduction in the compressive strength for the control concrete (CC) and for the concrete modified with styrene–butadiene dispersion (PC2). For the control concrete, the average value of this decrease was about 4%, while for PC2 concrete it was 12%. Such a significant strength reduction for PC2 concrete is connected with the meaningful increase in strength for the samples stored in de-mineralized water. Presumably, cement hydration reactions slowed down during the stage of maturation, and the following elongation may be caused by the presence of polymer membranes that penetrated the composite structure and hindered water migration.

However, the differences in the compressive strength levels for particular concretes stored for a standard time, equal to 1000 h, in crude-oil products were so slight that they may be ascribed to measurement variations. Presumably, the storage time for the tight concretes tested was too short to determine any further negative influence of crude-oil products in the environment on the strength parameters of these concretes.

**Figure 6:**
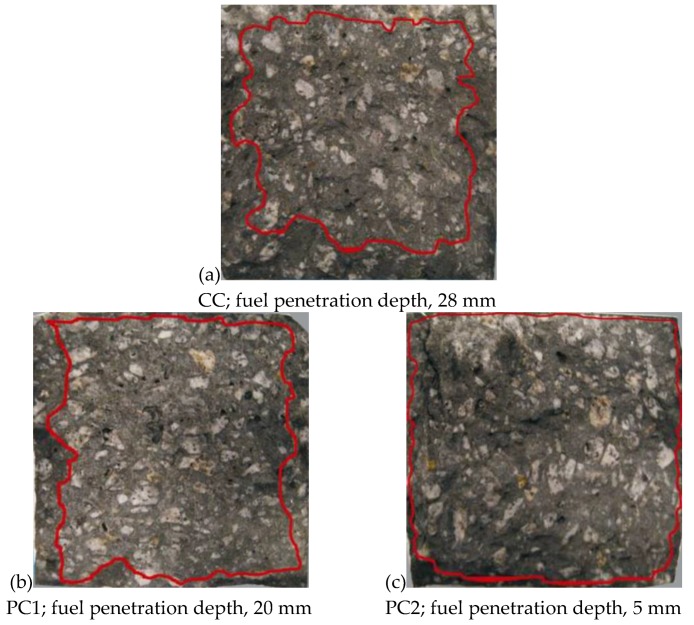
Fractures of samples stored for 1000 h in non-ethylated fuel together with the outlined fuel penetration depth.

Additional samples stored in non-ethylated fuel were split in order to determine the water penetration depth. The greatest depth for non-ethylated fuel, equal to 28 mm, was obtained for the control concrete (CC). The concrete modified with the vinyl-benzene and acrylic (PC1) polymer dispersion indicated a slightly lower depth equal to 20 mm. Non-ethylated fuel penetrated, to the smallest extent, the concrete modified with the styrene–butadiene dispersion (PC2), up to a depth equal to 5 mm. In Figure 6, the views of the fractures of these samples are presented together with an outline of the fuel penetration depth.

## 4. Conclusions 

Based on the conducted tests, the following conclusions may be drawn:

- Tests for 28-day strength indicated that concretes modified with polymer dispersions are characterized with a lower compressive strength than a control concrete (CC). For the PC1 modified concrete, the difference was almost 14%, while for PC2 it was 11%;
- Concretes modified with polymer dispersions, especially with the styrene–butadiene dispersion (PC2), indicated a smaller degree of liquid penetration into the samples. The penetration depth for non-ethylated fuel for PC2 concrete, after 1000 h of storage, was only 5 mm, while for the control concrete (CC) it was 28 mm. The water penetration depth determined after 28 days of concrete maturation was 60 mm for the control concrete and 15 mm for the PC2 concrete. There was a consistent general trend for the penetration depths for both liquids in the concrete, but the water penetration depths were greater, because pressure was applied on these samples;
- The compressive strength for the tested concretes determined after 1000 h of storage in crude-oil products was close to the 28-day strength. However, compared to the strength of the samples stored in de-mineralized water, there was a significant decrease in results, with the maximum difference equal to 12% for PC2 concrete.

The performed tests clearly demonstrate the beneficial effect of polymer dispersion on the increase of concrete durability and strength. Polymer films penetrate the cement composite structure, sealing the matrix as well as the contact zone: aggregate–cement paste.

## Figures and Tables

**Table 1:** Chemical composition and physical properties of the cement used in the tests.

Properties	Mean Value
**Chemical Properties**	
Loss on Ignition (%)	0.58
Insoluble Residue (%)	0.19
SO_3_ Content (%)	2.56
Cl^−^ Content (%)	0.007
Na_2_O_eq_ Content (%)	0.44
Al_2_O_3_ Content (%)	4.14
C_3_A Content (%)	1.42
C_3_S Content (%)	57.3
C_4_AF + 2C_3_A Content (%)	19.9
**Physical Properties**	
Initial Setting Time (min)	215
Final Setting Time (min)	270
2-day Compressive Strength (MPa)	16.8
28-day Compressive Strength (MPa)	49.7
Soundness (mm)	1.0
Specific Gravity (g/cm^3^)	3.2
Specific Surface Area According to Blaine (cm^2^/g)	3350

**Table 2:** Compositions of tested mixes for control and modified concretes.

Component	Composition (kg/m^3^)		
	CC	PC1	PC2
Cement	380	380	380
Water	133	133	133
Fine aggregate 0/2	720	720	720
Coarse aggregate 2/8	500	500	500
Coarse aggregate 8/16	700	700	700
Super-plasticizer	2.1	-	1.1
P1 (10% cem. mass)	-	38	-
P2 (10% cem. mass)	-	-	38

CC—Control Concrete; PC1—Concrete modified with a polymer dispersion of vinyl-benzene and acrylic; PC2—Concrete modified with styrene–butadiene dispersion.

**Table 3:** Test results for consistency and density of concrete mixes.

Type of Concrete Mix	Cone Fall (mm)	Consistency Class	Density (kg/m^3^)
CC	150	S3	2405
PC1	170	S4	2440
PC2	165	S4	2425

**Table 4:** Test results for 28-day compressive strength.

Type of Concrete	28-Day Compressive Strength (MPa)	
	f_ci_	f_cm_
CC	64.3	
	61.1	62.0
	60.6	
PC1	52.4	
	53.8	53.5
	54.3	
PC2	55.6	
	55.5	55.5
	55.4	

**Table 5:** Test results for water absorption and permeability for control concrete (CC) and for concretes modified with polymers (PC1 and PC2).

Type of Concrete	Water Absorption (%)		Water-Tightness Degree	Average Depth of Water Penetration (mm)
	WA_i_	WA_m_		
CC	4.0	4.0	W8	60
	4.1			
	3.9			
PC1	3.5	3.3	W8	30
	3.3			
	3.1			
PC2	2.5	2.5	W8	15
	2.4			
	2.6			

**Table 6:** Summary test results for strength of control concrete (CC) and polymer-modified concretes after storage in different environments.

Type of Concrete Mix	Mean Compressive Strength Determined after 1000 h of Storage in Liquids: f_c_ (MPa)			
	De-Mineralized Water	Diesel	Non-Ethylated Fuel	Mixture of Liquids
CC	67.2	63.0	64.5	65.5
PC1	54.5	53.1	48.0	54.0
PC2	62.8	56.5	57.6	52.9

## References

[1] Shahrabadi H., Sayareh S., Sarkardeh H. (2017). Effect of silica fume on compressive strength of oil-polluted concrete in different marine environments. China Ocean Eng..

[2] Anikiev V.V., Mishukov V.F., Moiseevsky G.N., Tkalin A.V. (1988). The Effect of Oil Films on Water Evaporation and Oxygen Content in Sea Water. GeoJournal.

[3] Majewski T., Niedostatkiewicz M. (2016). Repair of heavily oily concrete floor. Build. Rev..

[4] Pukhov I.E. (2001). Effect of Mineral Oil on the Reinforced-Concrete Floors of the Uglich and Rybinsk Hydroelectric Power Plants. Hydrotech. Constr..

[5] Ksit B. (2004). Changes in the mass and strength of oiled road concretes. Build. Mater..

[6] Linek M., Żebrowski W., Wolka P. (2016). Change of physical parameters and compressive strength of airport concrete under the influence of hydraulic mineral oil. Build. Mater..

[7] Pużak T. (2007). Concrete resistant to the influence of petroleum fuels and light liquids. BTA.

[8] Runkiewicz L., Konieczny K., Brzęk R. (2002). Changes in strength and deformability of oily concrete in the structure. Build. Rev..

[9] Błaszczyński T. (2003). Corrosion of concrete as a result of the interaction of hydrocarbon substances. Part 1. Mechanisms of concrete destruction by petroleum products. Corros. Prot..

[10] Błaszczyński T. (2003). Corrosion of concrete as a result of the interaction of hydrocarbon substances. Part 2. The method of assessing the degree of corrosion as a result of the interaction of hydrocarbon substances. Corros. Prot..

[11] Yurtdas I., Xie S.Y., Burlion N., Shao J.F., Saint-Marc J., Garnier A. (2011). Influence of chemical degradation on mechanical behavior of a petroleum cement paste. Cem. Concr. Res..

[12] Osuji S., Nwankwo E. (2015). Effect of Crude Oil Contamination on the Compressive Strength of Concrete. Niger. J. Technol..

[13] Błaszczyński T. (2011). Assessment of RC structures influenced by crude oil products. Arch. Civ. Mech. Eng..

[14] Salman A.A.A., Alghazali J.J.H., Alwash N.O.S. (2018). The effect of fibers on the properties of self-compacting concrete subjected to petroleum products. Int. J. Civ. Eng. Technol..

[15] Talib A. (2018). Influence of Oil Products on Strength and Durability of High Strength Latex Modified Concrete Coated by Epoxy. Kufa J. Eng..

[16] Abbas Z. (2017). Effect of Kerosene and Gas Oil Products on Different Types of Concrete. Int. J. Sci. Res..

[17] Jasim A.T., Faris A.J. (2017). Effect of Oil on Strength of Normal and High Performance Concrete. Al Qadisiya J. Eng. Sci..

[18] Svinstov A.P., Shambina S.L. (2018). Influence of viscosity of vegetable and mineral oil on deformation properties of concrete and cement-sand mortar. Constr. Build. Mater..

[19] Svinstov A.P., Nikolenko Y.V., Kharun M., Kazakov A.S. (2014). Effect of viscosity of petroleum products on deformation properties of concrete. Mag. Civ. Eng..

[20] Diab H. (2011). Effect of mineral oil on reinforced concrete structures. Part I. Deterioration of compressive strength. J. Eng. Sci..

[21] Diab H. (2012). Compressive strength performance of low- and high-strength concrete soaked in mineral oil. Constr. Build. Mater..

[22] Gruszczyński M. (2014). Resistance of Polymer Dispersion Additive Modified Concrete to Light Liquids Action. Adv. Mater. Res..

[23] Kameche Z.A., Ghomari F., Choinska M., Khelidj A. (2014). Assessment of liquid water and gas permeability of partially saturated ordinary concrete. Constr. Build. Mater..

[24] Abousnina R.M., Manalo A., Lokuge W. (2016). Physical and mechanical properties of cement mortar containing fine sand contaminated with light crude oil. Procedia Eng..

[25] Abousnina R.M., Manalo A., Lokuge W., Al-Jabri K.S. (2018). Properties and structural behaviour of concrete containing fine sand contaminated with light crude oil. Constr. Build. Mater..

[26] Wolicka D. (2010). Microorganisms found in crude oil and in reservoir waters. Oil Gas.

[27] Błaszczyński T. (2011). The influence of crude oil products on RC structure destruction. J. Civ. Eng. Manag..

[28] Lenart M. (2014). Influence of the composition of cement mortars modified by lime or polymers on the mechanical properties and microstructure of mortars. Key Eng. Mater..

[29] Łukowski P. (2008). Role of Polymers in Forming of Properties of Polymer-Cement Binders and Composites.

